# Psychometric properties of the embodiment scale for the rubber hand illusion and its relation with individual differences

**DOI:** 10.1038/s41598-021-84595-x

**Published:** 2021-03-03

**Authors:** Daniele Romano, Angelo Maravita, Marco Perugini

**Affiliations:** 1 Psychology Department, University of Milano-Bicocca, piazza dell’Ateneo Nuovo, 1, 20126 Milan, Italy; 2 Mind and Behavior Technological Center (MIBTEC), University of Milano-Bicocca, Milan, Italy; 3 Department of History, Society and Human Studies, University of Salento, Milan, Italy

**Keywords:** Psychology, Human behaviour, Perception

## Abstract

The Rubber Hand Illusion (RHI) opened the investigation of the sense of body ownership in healthy people. By putting in slight contrast vision touch and proprioception, healthy people embody a fake hand in one's body representation. The easiness of the procedure, typically measured with a set of questions that capture the subjective experience, favoured its blooming. However, validation studies of embodiment questionnaires are lacking, and the individual differences that contribute to the embodiment received little attention. In our study, 298 participants underwent an RHI procedure following both synchronous and asynchronous (control) visuo-tactile stimulations. The study had multiple aims: (a) to explore the psychometric structure of a 27-items questionnaire largely used in the literature; (b) to build a psychometrically efficient scale to measure embodiment-related phenomena; (c) to explore whether and how individual differences (empathy, self-esteem and mindfulness) are associated with the experience of illusion. We found a relatively simple structure consisting of three components: *embodiment* of the rubber hand, *disembodiment* of the biological hand, *physical sensations* experienced during the procedure. The scales designed were psychometrically reliable and theoretically meaningful, encompassing 18 of the original items. Finally, by adopting a network analysis approach, we found that the embodiment is directly related to empathy and self-esteem, while disembodiment and physical sensation are unrelated to individual personality traits. The study provides substantial evidence to use the embodiment scale as a standard questionnaire for future RHI studies. Additionally, the correlations with personality traits suggest that the embodiment induced by the RHI deeply integrates with the complexity of the individuals and their differences.

## Introduction

The sense that a part of the body belongs to oneself is typically referred to as "sense of body ownership". It is a core feature of humans life that is overall stable, but it can be subject to small or radical changes^1^. For instance, brain damage may deeply disrupt the feeling of ownership of one’s own body parts^2^. For a 100 years, body ownership has been investigated only in neuropsychological conditions, while it became of deep interest to experimental psychologists and neuroscientists only after the development of the Rubber Hand Illusion (RHI)^3^. The RHI was developed by Botvinick and Cohen. The sensation that an external object like a fake rubber harm belongs to oneself is generated by putting multiple senses in “slight contrast” (touch, vision, and proprioception)^3,4^.

In the RHI a visible fake hand is stroked synchronously with the real hand, which is hidden from view. A person embodies the fake hand in one’s body representation when certain parameters are satisfied. For example, the distance between the hands (i.e., less than around 30 cm)^5,6^, the anatomical plausibility of the fake limb (i.e., same body part oriented similarly to the real one)^7^, and the spatio-temporal congruency between visual stroke and touch events^3^ are essential parameters. The fake limb, to some extent, substitutes the real hand in the mental representation^1^. Crucially, the experience is variable across individuals. However, what determines individual differences in the embodiment experience has received little attention so far^8–11^.

In the last 20 years, the RHI procedure became a frequently used experimental procedure. From a PubMed search on “Rubber Hand illusion” (October 10th, 2020) 470 papers were found, with more than 30 papers published only in 2020.

Due to its versatility and efficacy, the RHI has fast grown as a standard procedure to investigate body ownership^4,12^. Recently, the RHI has also been used in clinical settings, to develop prostheses for amputees that could be more easily incorporated^13^ and alleviate neuropsychological symptoms after stroke^14^.

Almost every paper investigates the subjective experience induced by the RHI with a set of questions. Items are frequently created ad-hoc for each study. However, two sets of questions have been used multiple times by several research groups. First, the original 9-item questionnaire produced by Botvinick and Cohen^3^, which was never validated psychometrically to the best of our knowledge. Second, a set of 27 items that were analysed from a psychometric perspective by Longo et al. in 2008^15^.

The work by Longo et al. is the reference point for our study. The authors administered the RHI procedure (synchronous and asynchronous stroking conditions) to 131 participants. Participants had to answer a set of 27 items. The authors used a Principal Component Analysis (PCA) to investigate the underlying psychometric structure, finding two slightly different structures following the synchronous and the asynchronous stimulation procedures, respectively identifying a 4-component and a 5-component solution. The work was pioneering in this perspective, but a few problematic issues, such as the use of a relatively small sample and of an orthogonal rotation in the PCA, suggested that a further investigation could have been useful to consolidate the results.

### Aims of the study

In our study, there were three main aims.

First, we aimed to explore further the psychometric structure of the same 27 items in a larger cohort of participants (N = 298). Participants were native Italian speakers, providing a viewpoint from a different language and culture. We went further than merely replicating what Longo and colleagues did by changing a few key elements in the analysis procedure, exploring multiple component solutions, and using a much larger sample size.

Second, we aimed to build a psychometrically efficient Embodiment Scale (ES) to measure embodiment, starting from the original 27 items and identifying those that best capture the illusion.

Third, we aimed to explore whether individual differences impact the experience of the illusion. While it is common to observe that the RHI has huge variability, only a few studies attempted to understand the source of such variability^8–11^. We hypothesised that empathy, self-esteem, and mindfulness are potential individual traits that might be related to the RHI. Empathy is the trait related to how a person feels other's experiences. During the RHI, a person has to process a stimulation seen on another's hand. The degree to which such visual scene is internally shared by the onlooker, analogously to the empathic concern for someone else’s experience, may be critical to the occurrence of the RHI*.*

Furthermore, since sensory awareness has been linked to the RHI^8,11,16^, we investigated whether a questionnaire capturing the *mindfulness* attitude, i.e. the attitude towards analysing internal states generated by sensory experience, could capture the sensitivity to the RHI.

Finally, *self-esteem* reflects one's overall sense of self-worth, a trait that can potentially deviate upward every self-referred judgment, possibly including the reference of an alien body part to oneself.

The ES score was therefore correlated to the scores obtained in three questionnaires dedicated to measuring empathy (Interpersonal Reactivity Intex—IRI^17^), mindfulness (Five Facet Mindfulness Questionnaire—FFMQ^18^), and self-esteem (Rosenberg self-esteem scale—RSE^19^). To deepen the investigation of individual differences, we explored the relations of these constructs and RHI scores through a network analysis approach. A network is a model composed of nodes, representing entities, and a set of edges that connect the nodes, representing their relations. The use of networks leads to a more comprehensive, simultaneous picture of the direct and indirect relations characterising the whole set of variables^20^, going beyond the mere correlation of pairs of variables.

To achieve the study aims, we designed a series of analyses with specific goals summarised below (see “Methods” for a detailed description).

1. Exploration of the psychometric structure of the same 27 items.
  We first performed a Principal Component Analysis (PCA) of the 27 items of the questionnaire by Longo et al., collected after synchronous stimulation (*Best solution of component structure of the RHI*). Solutions were Oblimin-rotated, a key element that differentiates the current study from Longo's one. Indeed, by using Varimax rotation, Longo and colleagues forced the non-correlation between components, while the Oblimin allowed the components to correlate one with each other, which is arguably a very likely empirical possibility.
  Because we did not replicate in full the original solution of Longo and colleagues, we explored subsequent solutions (*Hierarchical emergence of embodiment structure in the RHI*) from the 3-component model (i.e., our best solution), up to a 7-component model (i.e., the most complex solution sustainable with our data). By adopting a Bass–Ackwards hierarchical procedure^21^, one can explore different granularity levels (or specificity) among the plausible component solutions. In this way, it is possible to understand the relation between broader components and more specific components that might emerge only when granularity is high. The Bass-Ackwards procedure works as both a control of the selected solution's quality and an exploration for the merits of potential alternative solutions.
  One can argue that a questionnaire measures a construct (e.g., the embodiment) rather than reflecting the participant's behaviour in a specific experimental condition (e.g., the synchronous stimulation) if the underlying structure of the items is similar in different conditions. Therefore, establishing similar structures for the synchronous and asynchronous conditions is essential to allow a meaningful comparison between the respective scores. Thus, we followed an exploratory PCA of the 27 items after asynchronous stimulation, with the Bass-Ackwards procedure on the same items, exploring solutions from the 3-component to a 7-component, as we did for the synchronous stimulation (*The component structure of embodiment in the control condition*).
2. The building of a psychometrically efficient scale to measure embodiment (*Embodiment Scale—ES*).
  We elaborated a restricted version of Longo's questionnaire by selecting only the items that work efficiently in measuring the experience elicited by the RHI (*Items reduction*).
  A PCA on the selected items was employed to test that the proposed scales are psychometrically adequate. Tucker's phi verified that the three-component solutions for synchronous and asynchronous stimulation were comparable. The structure's goodness was further ascertained by performing a confirmatory factor analysis (CFA) on the selected items.
  We calculated the scale scores averaging the values of the items loading on each component. Each scale's internal consistency was measured with Cronbach's alpha and McDonald's omega (*Psychometric properties of the Embodiment Scale and its subscales*).
  Then, we matched synchronous and asynchronous conditions on the average level of every subscale with three paired samples t-tests to verify which subscale is sensitive to the multisensory discrepancy (*Measure of the RHI with the ES*).
3. Individual differences in RHI effect.
  We first calculated the effect of the RHI. The effect can be defined as the difference between the synchronous and asynchronous stimulations for each subscale. Then, we correlated delta scores with the questionnaires' scores for empathy, mindfulness and self-esteem, using a network analysis approach (*Network Analysis of ES and individual differences*), a method useful to explore the correlations among a large set of intercorrelated variables. We used a Gaussian Graphical Model (GGM) to estimate a conservative and parsimonious network with robust estimates^22,23^.

## Results

### Exploration of the psychometric structure of the same 27 items

#### Best solution of component structure of the RHI

**Figure 1:**
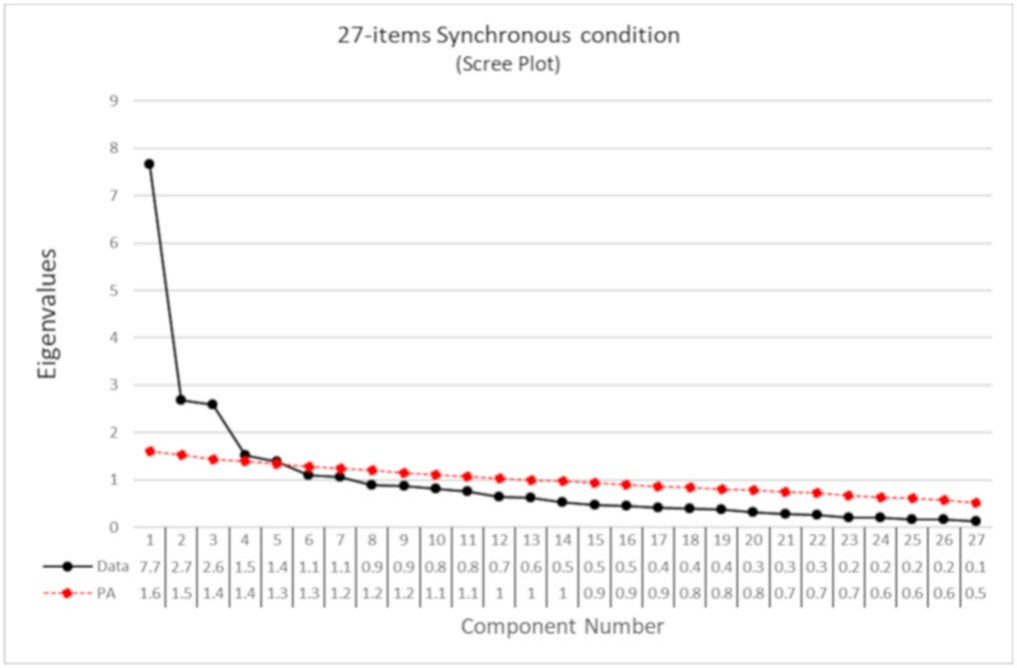
Scree-plot of the PCA on the 27 items after synchronous condition. The eigenvalues are reported in the table below the graph. The black dots represent the eigenvalues of each component in decreasing order calculated on the real data. The red line shows the simulated data of the parallel analysis. Components above the red line are those indicated by the parallel analysis. The gap after the third component in the scree-plot suggests considering a three-components solution.

Seven components had eigenvalues > 1. The parallel analysis suggested to extract 5 components, and the Velicer's Minimum Average Partial (MAP) suggested 3 components. The scree plot (Fig. 1) suggested a clear gap after the third component and a smaller gap after the fifth component. Based on these criteria, as well as on an inspection of the content of the items, the best solution was the three-components.

The three-components solution explains 48% of the variance, with the first and the second factors that are noticeably correlated (0.37). The three components can be interpreted clearly. The first component captures all the items about the fake hand's embodiment (items 1:11) and can be called "*embodiment*". The second component is loaded by items related to the control of one’s real hand (items 12:18), therefore unifying the components of Longo’s solution named "loss-of-own-hand" and "movement", in a single component that we propose to name "*disembodiment*". Finally, the third component is primarily loaded by items 22 and 23 concerning the pleasantness/unpleasantness of touch, with weaker loadings from items 24 to 27 that refer to other tactile experiences. All together, these items referred to the physical sensations felt on one's real hand, that we propose to name "*physical sensations*".

While most items clearly load on their respective component, a few items show some cross-loadings (Table 1).

**Table 1:** Matrix of loadings, variance explained by the components, and their correlations.

During the block…	Items no.	TC1 (embodiment)	TC2 (disembodiment)	TC3 (physical sensations)
…it seemed like I was looking directly at my own hand, rather than at a rubber hand	**1**	**0.79**	− 0.03	0.05
…it seemed like the rubber hand began to resemble my real hand	**2**	**0.71**	0.04	− 0.07
…it seemed like the rubber hand belonged to me	**3**	**0.85**	0.03	0.03
…it seemed like the rubber hand was my hand	**4**	**0.88**	0.01	0.00
…it seemed like the rubber hand was part of my body	**5**	**0.87**	− 0.03	0.05
…it seemed like my hand was in the location where the rubber hand was	**6**	**0.71**	0.02	− 0.09
…it seemed like the rubber hand was in the location where my hand was	**7**	**0.42**	0.15	− 0.12
…it seemed like the touch I felt was caused by the paintbrush touching the rubber hand	**8**	**0.68**	− 0.08	0.19
…it seemed like I could have moved the rubber hand if I had wanted	**9**	**0.74**	0.02	− 0.03
…it seemed like I was in control of the rubber hand	**10**	**0.74**	0.06	− 0.05
…it seemed like my own hand became rubbery	11	0.44	0.33	− 0.23
…it seemed like I was unable to move my hand	**12**	0.09	**0.67**	− 0.10
…it seemed like I could have moved my hand if I had wanted	13	0.28	− 0.63	0.03
…it seemed like I couldn't really tell where my hand was	**14**	0.08	**0.49**	0.02
…it seemed like my hand had disappeared	**15**	0.08	**0.63**	0.00
…it seemed like my hand was out of my control	**16**	0.01	**0.80**	− 0.01
…it seemed like my hand was moving towards the rubber hand	**17**	0.05	**0.63**	0.17
…it seemed like the rubber hand was moving towards my hand	**18**	0.01	**0.73**	0.08
…it seemed like I had three hands	19	0.01	0.33	0.31
I found that experience enjoyable	20	0.24	− 0.19	0.04
I found that experience interesting	21	0.47	− 0.31	0.08
…the touch of the paintbrush on my finger was pleasant	**22**	0.07	− 0.04	**0.87**
…I had the sensation of pins and needles in my hand	**23**	0.11	0.01	− **0.82**
…I had the sensation that my hand was numb	24	0.26	0.31	− 0.42
…it seemed like the experience of my hands was less vivid than normal	25	0.13	0.31	0.36
…I found myself liking the rubber hand	26	0.35	0.16	− 0.33
…it seemed like I was feeling the touch of the paintbrush in the location where I saw the rubber hand being touched	27	0.17	0.27	0.67
	Eigenvalues	6.53	3.83	2.58
	Proportion Var	0.24	0.14	0.10

	Component correlations			
		TC1	TC2	TC3
"Embodiment"	TC1			
"Disembodiment"	TC2	0.37		
"Physical sensations"	TC3	0.03	− 0.02	

Among the questionnaire items reported in the first column, those in bold are included in the embodiment Scale. See also the Supplementary Material for a model form of the embodiment Scale.

#### Hierarchical emergence of embodiment structure of the RHI

We explored subsequent solutions from the 3-component model, which corresponds to our best solution, up to a 7-component model, which corresponds to the most complex sustainable solution (justified by eigenvalues > 1, see below), using a Bass–Ackwards procedure^21^. The detailed description of each solution is reported in the Supplementary Material 1.

**Figure 2:**
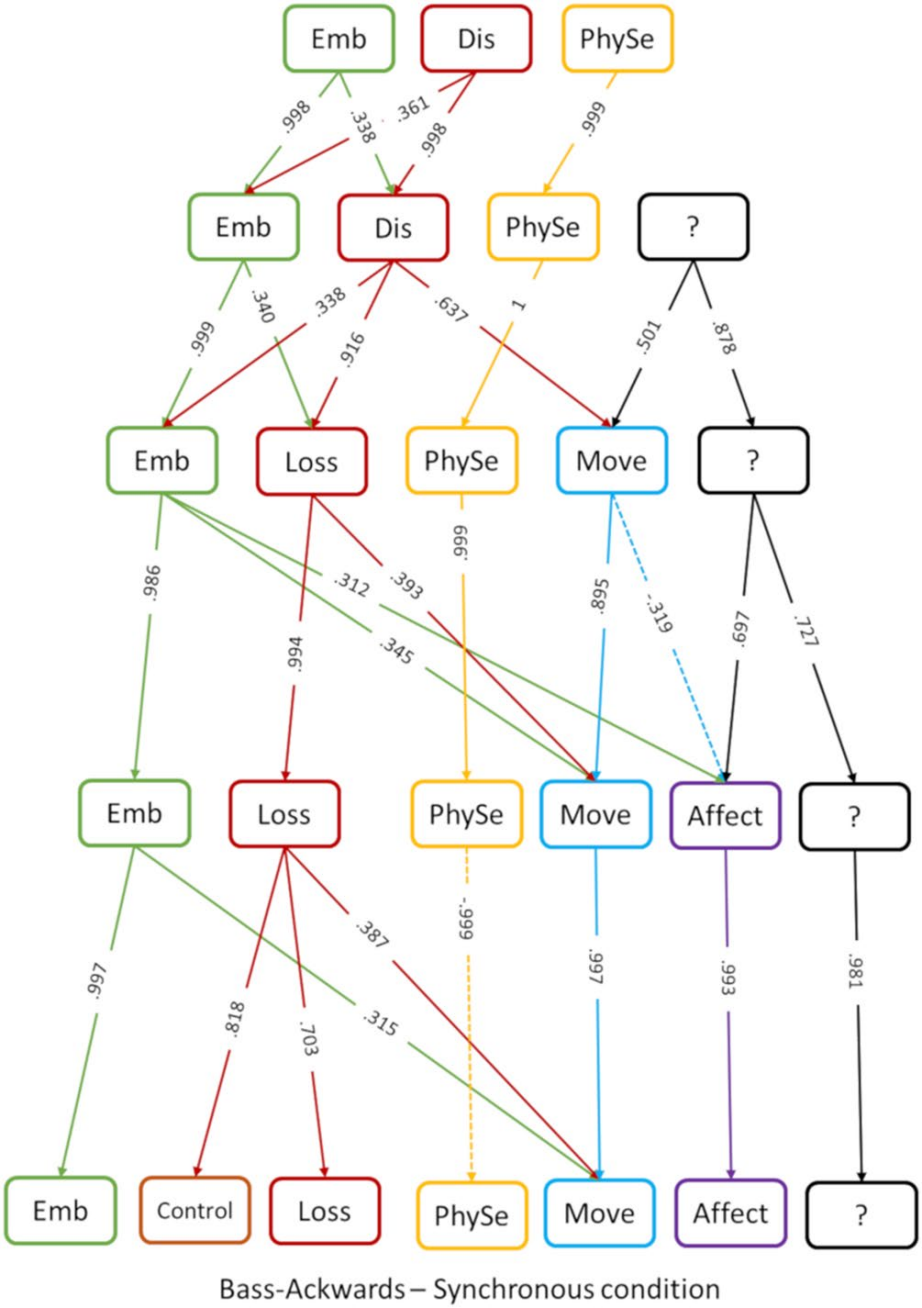
Results of the Bass–Ackwards procedure on the items after synchronous stimulation. The correlations between the components of different solutions are represented. Negative correlations are reported with dash lines, while positive correlations are in solid lines. Components’ names are abbreviated: *Emb* embodiment, *Dis* disembodiment, *PhySe* physical sensations, *Loss* loss-of-own-hand, *Move* movement, *Affect* affect towards the general experience, *Control* control on own hand actions, *?* undefined component. Colours are used to help the readability of the graph.

Correlations between components scores of different solutions are depicted in Fig. 2.

The first component remains substantially the same across all solutions with a correlation > 0.95 with the homologous component of the next solution. We never clearly recovered the three subcomponents defined by Longo^15^. Only from the six-component solution something similar to the Location component emerges. However, it is very unclear as only item 7 and 19 unequivocally loaded on it, while the theoretical solution would have pointed to item 6, 7 and 8. Interestingly, the disembodiment component splits in the two-component observed by Longo' study, increasing the complexity of the structure. They emerge as independent components in the five-component solution.

Notably, we identified a component about physical sensations experienced on one's hand already in the three-component solution. This component remains unaffected by the increasing complexity with correlations > 0.98 from one solution to another. This component is similar, although not identical, to the one described by Longo as "deafference". Interestingly, deafference emerged only following asynchronous stimulation in their study, while we have clear evidence of this component about physical sensation in the basic structure of the synchronous component solution.

#### The component structure of embodiment in the control condition

**Figure 3:**
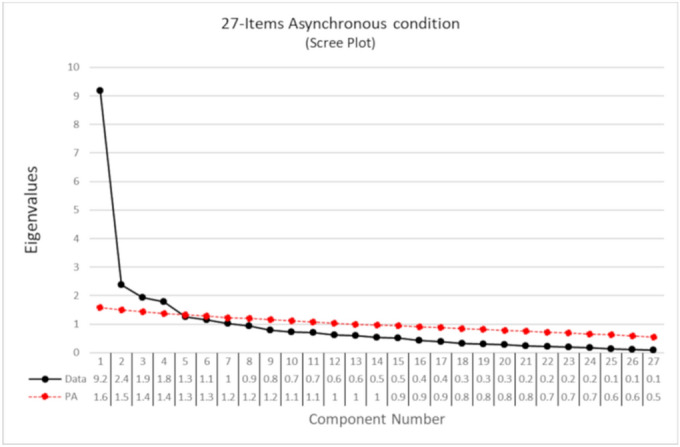
Scree-plot of the PCA on the 27 items after asynchronous condition. The eigenvalues are reported in the table below the graph. The black dots represent the eigenvalues of each component in decreasing order. The red line shows the simulated data of the parallel analysis.

The exploratory PCA of the 27 items after the asynchronous condition showed seven components with eigenvalues > 1. The parallel analysis suggested 4 components. MAP suggested 4 components. The scree plot (Fig. 3) suggested a gap after the first and the fourth component. The suggested solution, independent from synchronous stimulation, would seem, therefore, a four-component solution.

The detailed description of each solution obtained with the Bass–Ackwards produces is reported in the Supplementary Material 1.

**Figure 4:**
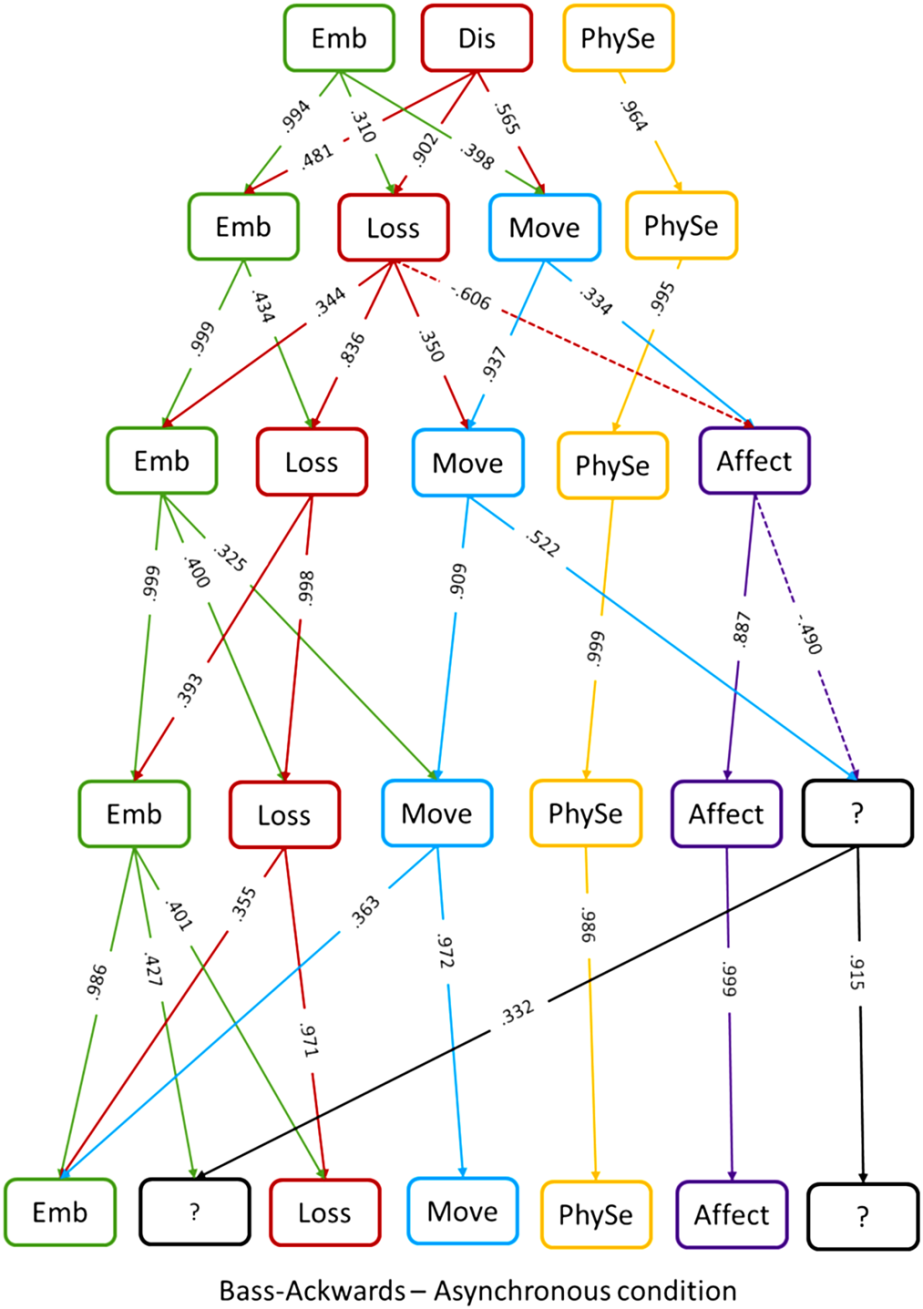
Results of the Bass–Ackwards procedure on the items after asynchronous stimulation. The correlations between the components of different solutions are represented. Negative correlations are reported with dash lines, while positive correlations are in solid lines. Components’ names are abbreviated: *Emb* embodiment, *Dis* disembodiment, *PhySe* physical sensations, *Loss* loss-of-own-hand, *Move* movement, *Affect* affect towards the general experience, *Control* control on own hand actions, *?* undefined component. Colours are used to help the readability of the graph.

Correlations between components of subsequent solutions are represented in Fig. 4.

Also in the asynchronous condition, we were unable to distinguish any subcomponent of embodiment (ownership, location or agency). The correlations between components showed that the component embodiment and physical sensations remain constant in all solutions. The emergence of the components up to the five-component solution appeared easy to interpret. In contrast, the six and seven components solutions seem to be a not good explanation of our data resulting in almost meaningless components. In the five-component solution, the component affect emerges almost as a new component and is only slightly correlated with the movement component of the four-component solution. The most interesting issue is what happens when going from the three- to the four-component solution. While two components remained basically identical, we observed a split of the component disembodiment in two sub-components, one related to movement sensations and one to the experience of losing one's hand. Therefore, the four-component solution does not introduce anything radically different from the three-component solution, but it simply offers a finer distinction of one of the three components. Recall that a meaningful comparison of scores across conditions requires similarity among the component structures. Therefore, we think that overall, the three-component solution should be the preferred one, also in this case. In other words, the comparability of the component solutions between the synchronous and asynchronous conditions is a much greater benefit than the small costs associated with the loss of a finer-grained distinction between two sub-components.

### The construction of a psychometrically efficient scale to measure embodiment (Embodiment Scale—ES)

#### Items reduction

From the original three-component solution on the synchronous condition, we selected the items that are good markers for a component. Thus, we selected the items with a primary loading > 0.4 and any secondary loading < 0.25 (1:10, 12, 14:18, 22, 23).

**Figure 5:**
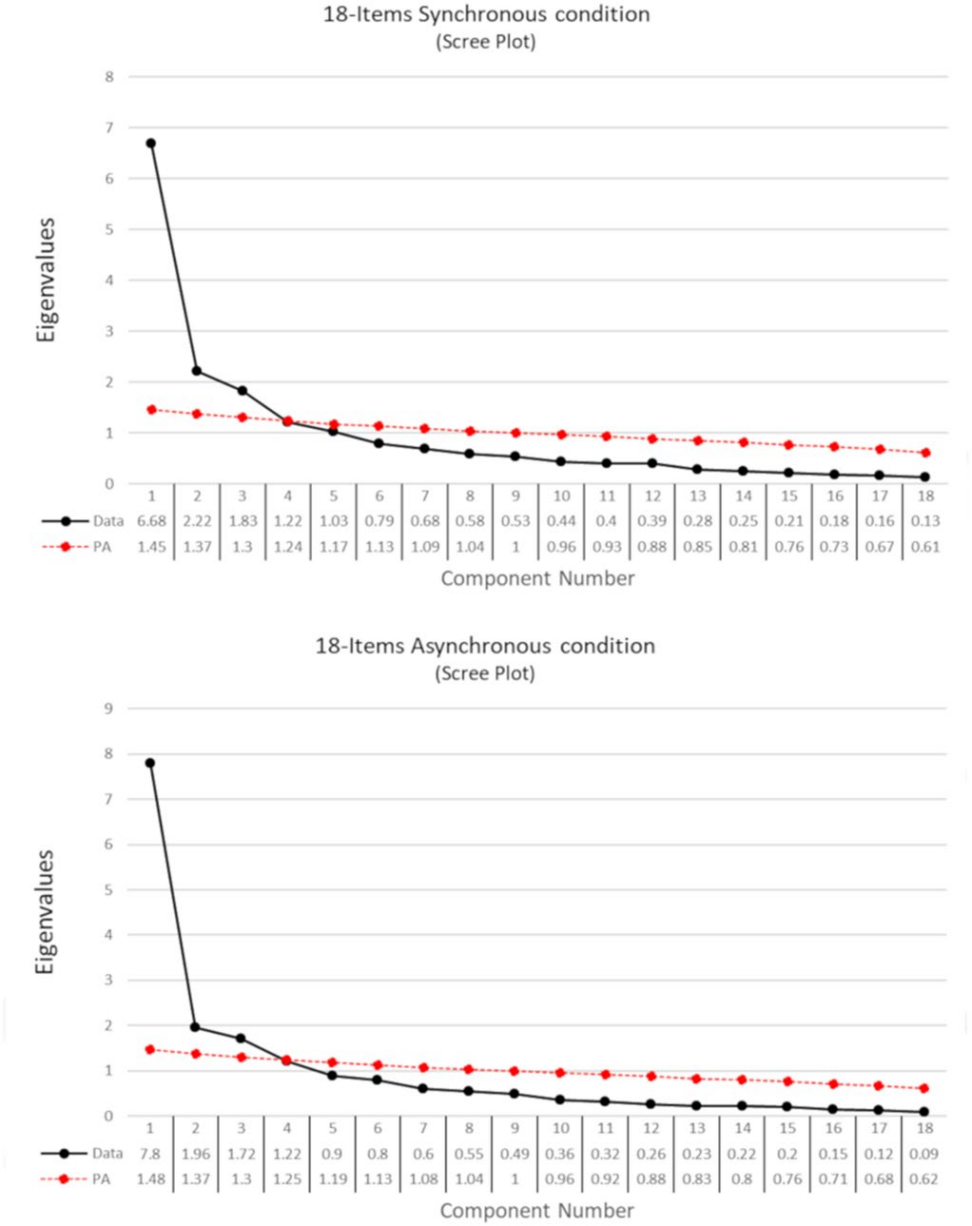
Scree-plots of the PCA on the 18 items selected for the embodiment scale. The upper panel referred to the synchronous stimulation condition, and the lower panel referred to the asynchronous stimulation condition. The eigenvalues of each component are reported in the tables below the respective graph. The black dots represent the eigenvalues of each component in decreasing order. The red line shows the simulated data of the parallel analysis.

The results of the PCA on the selected 18 items show that five components have eigenvalue > 1. Parallel analysis suggests selecting three components, and MAP suggests selecting two. The scree-plot shows a clear gap between the third and fourth component (Fig. 5 upper panel). The three components are clearly interpretable. Component 1 refers to embodiment sensations (1:10), Component 2 refers to disembodiment sensations (12, 14:18), Component 3 refers to physical sensations (22, 23).

The PCA on the same items after asynchronous stimulation shows that four components have eigenvalue > 1. Parallel analysis suggests selecting three components, and MAP recommends six. The scree-plot shows a clear gap between the third and fourth component (Fig. 5 lower panel). The three components are clearly interpretable and similar to the synchronous solution. Component 1 refers to embodiment sensations (1:10), Component 2 refers to disembodiment sensations (12, 14:18), Component 3 refers to physical sensations (22, 23) (see Table 2).

**Table 2:** Matrix of standardised loadings of the CFA of the 18 selected items.

Items	Synchronous			Asynchronous		
	Embodiment	Disembodiment	Physical sensations	Embodiment	Disembodiment	Physical sensations
1	0.74			0.86		
2	0.70			0.79		
3	0.89			0.93		
4	0.92			0.95		
5	0.89			0.91		
6	0.67			0.69		
7	0.41			0.58		
8	0.56			0.60		
9	0.65			0.63		
10	0.66			0.61		
12		0.64			0.70	
14		0.56			0.62	
15		0.68			0.74	
16		0.82			0.90	
17		0.44			0.38	
18		0.52			0.39	
22			0.86			0.84
23			0.84			0.88

	Embodiment	Disembodiment	Physical sensations	Embodiment	Disembodiment	Physical sensations
**Factor correlations**						
Embodiment	(0.92)			(0.94)		
Disembodiment	0.43	(0.80)		0.50	(0.81)	
Physical sensations	0.01	0.06	(0.83)	− 0.24	− 0.21	(0.85)

On the component correlations diagonals, Cronbach's alpha of the scale is reported in the parenthesis.

The three-component solution on the selected items of synchronous stimulation was compared with the three-component solution obtained with asynchronous items to verify that it is satisfactory also for the control condition.

In fact, an important issue was to formally establish the similarity between the two structures. To this end, we calculated the Tucker's phi. All three components showed similarity across conditions with the values of the congruence coefficients ≥ 0.94 (Component 1—embodiment = 0.99; Component 2—disembodiment = 0.99; Component 3—physical sensations = 0.94). These results demonstrate that the three-component solution for asynchronous stimulation replicates the structure of synchronous stimulation, which is essential for a meaningful comparison of scale scores across conditions.

As a further confirmation, the CFA showed a good fit^24–26^ for the three-factor solution for both the synchronous and asynchronous stroking conditions. Both the incremental (CFI and TLI ≥ 0.95) and the absolute (RMSEA and SRMR < 0.08) fit indices support the goodness of the three-factor solutions (Table 3).

**Table 3:** Fit indices of the CFA are reported for both the synchronous and asynchronous conditions.

	χ^2^	RMSEA					Incremental fit indices	
		RMSEA	90% CI—lower	90% CI—upper	p value	SRMR	CFI	TLI
Synchronous	262.81	0.059	0.049	0.069	0.073	0.060	0.956	0.948
Asynchronous	280.59	0.063	0.053	0.073	0.019	0.063	0.960	0.952

All indices support the tested solution with three factors.

#### Psychometric properties of the Embodiment Scale and its subscales

Scales scores have therefore been calculated for the three sub-scales averaging the items of each scale, reverting items when necessary (e.g. Item 22).

Internal consistency of all three sub-scales is very good for both conditions. In the synchronous stimulation condition, Cronbach's alpha for the embodiment subscale is.92, while for disembodiment and physical sensation has values of 0.80 and 0.83, respectively. McDonald's omega was also good (embodiment = 0.92; disembodiment = 0.80; physical sensations = 0.84). Internal consistency of the asynchronous scales is substantially equivalent (Cronbach’s alpha: embodiment = 0.94; disembodiment = 0.81; physical sensations = 0.85; McDonald’s omega: embodiment = 0.94; disembodiment = 0.82; physical sensations = 0.85). The three scales have all good reliabilities (i.e., internal consistency) and are therefore adequate to measure the three components of the RHI experience.

#### The measure of the RHI with the ES

**Figure 6:**
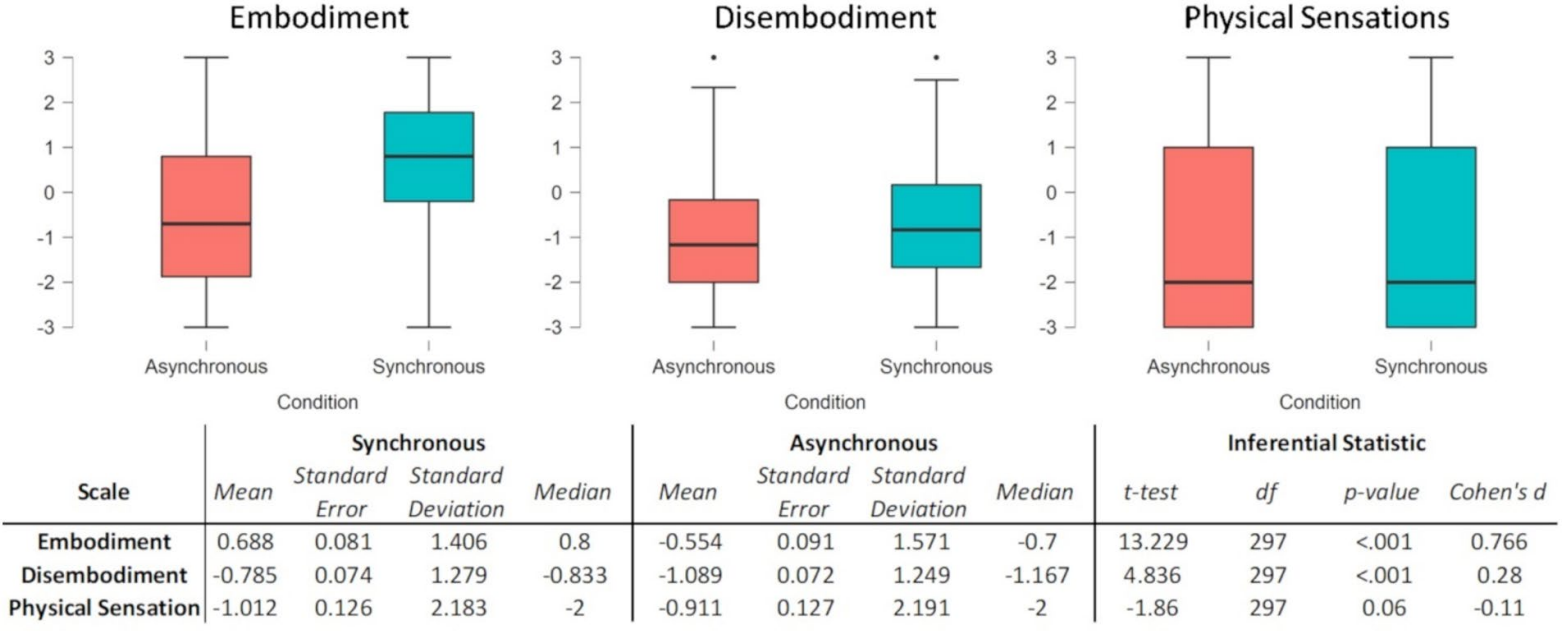
Scale scores are depicted in the graphs and reported in the associated table in the lower part of the figure. The graphs are boxplots where the thick lines represent the medians, the boxes represent the interquartile ranges (25°–75°), and the whiskers represent minimum and maximum. The associated table reports the descriptive statistics for the synchronous and asynchronous conditions, and the paired samples t test results of the Synchronous Vs Asynchronous match. Cohen's *d*_*z*_ was used to report the effect size.

Descriptive statistics show the different levels of each sub-component achieved during both the synchronous and asynchronous conditions. It is remarkable that the embodiment subscale, following the synchronous stimulation, is the only one with average positive values (Fig. 6). We then used a series of paired samples t-tests to verify any difference between the synchronous and asynchronous conditions in the three subscales. The t tests (Fig. 6) show a difference in the embodiment and disembodiment subscales with higher values following synchronous compared to asynchronous stimulation. Embodiment subscale has a larger effect size than disembodiment (almost three times larger), suggesting that this subscale is the most sensitive to the difference between synchronous and asynchronous stimulations.

The physical sensation did not show a significant result, although it was close to the threshold of 0.05 (p value of 0.06). Notably, the effect size is small (Cohen's *d*_*z*_ = *− *0.11), reflecting higher values for the asynchronous relative to the synchronous stimulation.

### Individual differences in RHI effect

We observed significant correlations between the embodiment subscale and the subscales measuring empathy (Table 4). Specifically, the fantasy scale has the strongest correlation. The negative sign (i.e., scores go in the opposite direction) suggests that the lower the score in fantasy, meaning that the less a person can put oneself in book or film characters, the stronger the embodiment experience. Other significant correlations were observed for perspective-taking and personal distress. Perspective-taking had a positive sign (i.e., scores go in the same direction) suggesting that the more a person is able to take the viewpoint of another person, the stronger the embodiment. The personal distress has a negative sign suggesting that the less a person is able to manage stressful situations, the stronger the embodiment experience. We also found a substantial correlation between the self-esteem questionnaire and the embodiment with a positive sign, suggesting that the more a person reports high self-esteem, the higher the rubber hand's embodiment. Notably, correlations here are calculated on the delta score between synchronous and asynchronous values, thus capturing the effect of the RHI net of each person's response set. Correlations were limited to the embodiment subscale, and none of the Mindfulness subscales correlated with any other scale.

**Table 4:** Correlation matrix between the RHI effect measured by the Embodiment Scale (synchronous–asynchronous) and the personality traits.

Scale	Subscale	Embodiment	Disembodiment	Physical sensations
IRI	Perspective taking	0.188**	0.088	0.061
	Fantasy	− 0.435***	− 0.116	0.085
	Empathic concerns	0.097	0.03	− 0.015
	Personal distress	− 0.173**	− 0.054	− 0.070
FFMQ	Observe	− 0.034	0.121	− 0.140
	Describe	0.006	0.024	0.044
	Awareness	− 0.046	0.051	− 0.024
	Non-judge	− 0.108	0.078	0.006
	Non-react	− 0.004	0.072	− 0.015
	Total	− 0.058	0.098	− 0.030
RSE	Self-esteem	0.424***	0.170*	− 0.112

*p value < 0.05; **p value < 0.01; ***p value < 0.001.

Importantly, correlations show a simple association between two variables. However, when the analysis focuses on many variables, potentially inter-correlated, there is a risk of overinterpreting results. A network analysis approach reduces this problem by analysing partial correlations and driving to more reliable conclusions^20^.

#### Network analysis of ES and individual differences

**Figure 7:**
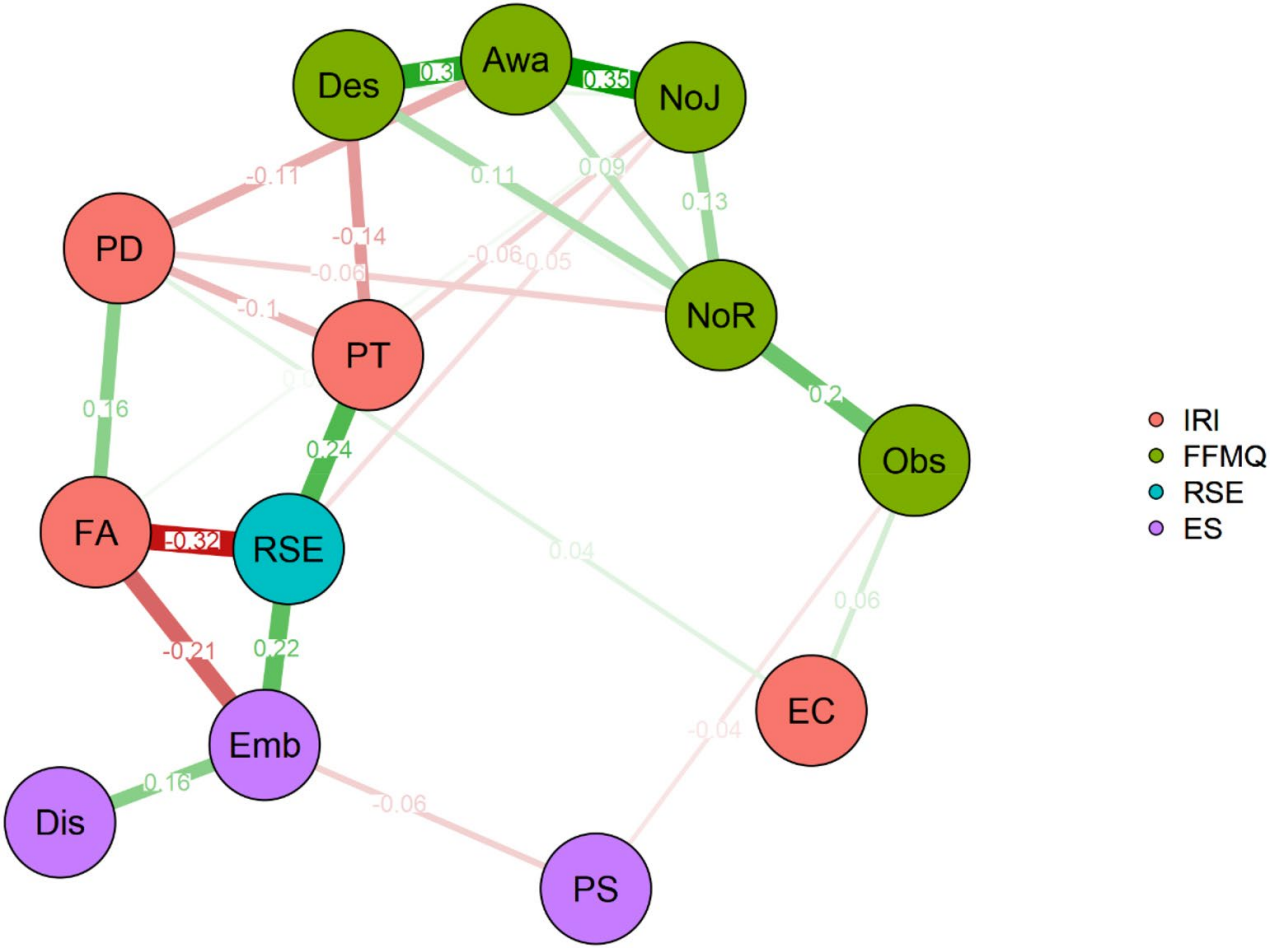
Network analysis results. Each node represents a different variable. The colour scheme helps to identify the nodes coming from different questionnaires. Edges show the association left between any two variables after conditioning on all other variables. Red edges identify negative relations. Green edges show positive relations. Edge labels report the exact value of the relation estimated by the GGM. The edge thickness and colour saturation is related to the edge value and helps in popping out the strongest relations. *ES* Embodiment Scale): *Emb* embodiment, *Dis* disembodiment, *PS* physical sensations. *IRI* (Interpersonal Reactivity Index): *FA* fantasy, *EC* empathic concerns, *PD* personal distress, *PT* perspective taking. *FFMQ* (Five Facet Mindfulness Questionnaire): *Obs*  observe, *NoR* non-react, *NoJ* non-judge, *Awa* awareness, *Des* describe, *RSE* Rosenberg self-esteem scale.

The network analysis (Fig. 7) showed a complex set of connections between the different facets of the measured personality traits. By focusing our attention to the ES subscales, it is worth noting that the edges that survive to the partialisation and the regularisation operated by the GGM identify a clear situation. Indeed, we can observe that only the fantasy scale and the RSE remained connected to the embodiment scale. This result has two major implications: first, the relations between the embodiment, the fantasy scale and the self-esteem are direct and consistent; second, the correlations between personal distress and perspective taking with embodiment (observed with simple correlations) do not translate into a direct effect. In fact, the links between both dimensions and the embodiment scale are indirect and passing through fantasy for personal-distress, and self-esteem for perspective-taking. Both fantasy and self-esteem, therefore, represent a bridge connecting the measured personality aspects and individual differences in the strength of the embodiment induced by the RHI.

An additional observation is that we do not observe any additional relation for the disembodiment and physical sensation facets when considering only the direct connections. There is one exception concerning the relation between physical sensations of the ES and the observe scale of the FFMQ, which however is of negligible size (− 0.04).

## General discussion

In the present work, we first wanted to characterise the different RHI experience components in a relatively large sample of Italian speakers. Second, we wanted to investigate if individual differences impact the RHI, focussing on empathy, mindfulness, and self-esteem constructs. Starting from the 27 items of the questionnaire proposed by Longo and co-workers, the present work indicates that a selection of such items, that we organised in a new Embodiment Scale (ES), may optimally describe embodiment experience in the RHI^15^. ES has the potential to become a standard questionnaire for future studies in the RHI (See the Supplementary Material 1 for a model of the Embodiment Scale and its scoring).

Longo et al.^15^, identified four main components following the synchronous stimulation. The first component is the embodiment that resumes all the items about the fake hand. Embodiment splits in three in a second stage analysis, differentiating between items related to the sense of ownership, the sense of agency, and the colocation of the real and fake hands. The other components collect items about the sensation of losing one's hand (loss of hand), items related to the sensation that hands were moving (movement), and items related to affective sensations experience during the procedure (affect). In the asynchronous condition, a deafference component adds to the previous one, collecting items about physical sensations like numbness.

Our best solution is simpler. Our results suggest that three components capture the underlying structure of the items measuring the RHI. The first component refers to the embodiment of the fake hand, and overlaps with the embodiment component of Longo et al.^15^. The second one collects all the items about the experience felt on one's hand, and it is a sum of the components loss of own hand and movement by Longo et al.^15^. We may call this component disembodiment, a concept theoretically relevant in the context of body representation^27^. Experimental evidence suggests that, together with the inclusion of the fake hand in body representation, participants can also decrease the embodiment's experience towards the real limb^28,29^. The RHI can also drive to physiological changes that suggest that when the fake hand is embodied, the real hand is disembodied^30–32^. The idea is that the body representation is keen to include external objects in body representation. Still, the body's structural representation is less malleable, so that, for example, the embodiment of multiple rubber hands is prevented^33^, and the structural constraints must be respected. From this perspective, the embodiment of a fake hand should drive to the real one's disembodiment. A corollary theoretical perspective is that body representation uses probabilistic representations of the hands instead of a dichotomous mine/not-mine distinction. With this perspective, multiple hands can be represented simultaneously in one's body representation with different levels of probability to belong to the self. From this perspective, ownership itself would not be an all or nothing property, but it would be a probabilistic property^2^, and the way we measure it can determine specific experimental effects. As a matter of fact, embodiment and disembodiment were correlated in our solution. This was not previously observed because of a technical aspect. In Longo's work, the authors adopted a varimax rotation which is orthogonal, forcing the components to have zero correlations. By using an Oblimin rotation, we allowed correlations between components, capturing an additional feature of the RHI experience. The RHI has been associated with physiological changes related to the disembodiment of one's body^30,32^. The correlation that we observed provides an empirical bridge between physiological changes associated with one's body's disembodiment and the embodiment of the fake body part.

The third component is narrow, with a few items that have strong loadings on it and only two crucial items that identify the component. The items referred to the physical sensations felt during the experience and to some extent, is similar to the deafference component found in Longo's work following asynchronous stimulation. Notably, although it is mainly composed of two items, this component was very consistent in our solution being detectable in both conditions of stimulations and at every level of solution complexity explored during the Bass-Ackwards procedure. The CFA models further support the three-factor solutions, with most of the fit indices converging in supporting the item selection's goodness and the solution proposed.

The three-component solution that we identified did not replicate in full the one by Longo. However, exploring more complex solutions with the Bass–Ackwards , we could identify the Loss-of-own-hand and the Movement components (see Supplementary Material 1 for a more detail analysis and discussion of the bass-ackwards solutions). The level of the details (e.g., number of components) depends on a sort of zooming in or out. As shown by the Bass-Ackwards results, Movement and Loss-of-own-hand are branches of the more general component disembodiment. A similar argument can also be made for the embodiment component and its components (see Supplementary Material 1 for a more detail analysis and discussion of the embodiment component composition). We want to point out that the study by Longo and colleague^15^ is a reference for our research. It inspired us, and we adopted the set of questions used in that work.

A second relevant achievement of the current study regards the development of the ES, which is an important step in measuring the RHI effect. First, the adoption of this selection of items, which is based on solid psychometric analysis, will improve the comparability across future studies. The subscales individuated are theoretically relevant, show a very good internal consistency, and are supported by the component analysis run on one of the largest sample sizes in the RHI literature.

The three scales show different sensitivity to the stimulation conditions. The embodiment and the disembodiment scales showed higher values in the synchronous than the asynchronous condition. The same conclusion cannot be drawn on the physical sensation which did not show a clear statistical significance, and remarkably, if any the difference goes in the direction of a stronger effect in the asynchronous than the synchronous. Notably, the effect size is larger for the embodiment than the disembodiment (almost three times) suggesting that the first ten items are the most sensitive to the RHI experience's specificity.

The ES also led to individuate how individual differences impact the RHI. We calculated the effect of the RHI as a difference between synchronous and asynchronous conditions. The RHI effect correlated with the measures of empathy. The embodiment subscale negatively correlated with the fantasy scale, and the personal-distress. It also positively correlated with the perspective-taking. The RHI also showed a positive correlation with the self-esteem scale. Notably, the network analysis is a method that can detect direct and indirect relations between a set of variables. Using this method, we have been able to identify that the direct relations are those left between the embodiment subscale, the fantasy scale (negative relation) and the self-esteem (positive relation). The other relations did not translate into direct links but were mediated by the two dimensions above that acted as bridges to the embodiment subscale.

In particular, the positive correlation between self-esteem and embodiment suggests that the higher one’s overall sense of self-worth, the higher the fake hand's embodiment, a result in line with the idea that self-esteem deviates upward every self-referred judgment. The fantasy scale is about the identification of oneself with fictitious characters. The correlation suggests that the less a person is able to identify oneself with fictitious characters of books and movies, the stronger the RHI effect. The network analysis shows a pattern of a few specific connections between the ES and the personality traits. Indeed, mindfulness was not correlated to any aspect of the RHI, except for a direct very small relationship between the observe scale and the physical sensation. Disembodiment was not directly connected to any personality trait node.

A previous study investigated the role of individual differences in the RHI. In that study, the RHI was associated with the sensory suggestibility, a personality trait related to how individuals react to sensory information^8^. Sensory suggestibility and mindfulness are constructs at least in part overlapping. Thus, we compared their results with what we observed in our data. Mindfulness did not show a consistent relation to the RHI nor as simple correlations neither as direct links in the network. Note that the methodological approach was very different from ours. Marotta et al.^8^ split their seventy participants in high/low sensory suggestibility, and then they compared the two groups on nine independent items about the illusion. They found an effect only on the ownership item. In our opinion, the method we adopted is more suitable for investigating individual differences. The RHI effect was measured with a questionnaire supported by psychometric evidence, and the effect of individual differences has been assessed with correlations and a network on much larger sample size. Crucially, the FFMQ and the sensory suggestibility scale, although shares some aspects, are built to measure slightly different constructs. So, it is genuinely possible that sense of ownership is related to sensory suggestibility. Still, the more general trait of embodiment is not related to the sensory awareness as measured with the FFMQ.

One limitation of this study is that it did not include behavioural counterparts of the RHI. Experimental evidence suggests that the subjective experiences captured with the questionnaires and the behavioural effects induced with the RHI (e.g., the proprioceptive drift) measure different facets of the illusion^34^. Thus, while our data suggest a relation between the subjective experience of embodiment and the individual differences, we cannot generalise the conclusion to all aspects of the RHI. It is therefore unclear whether the perceptual effects are sensitive to differences in personality traits. Future studies may focus their attention on this particular aspect.

The idea that individual differences can impact the RHI parallels the investigation of the embodiment in psychopathological conditions. It was indeed found that schizophrenia modulates the embodiment experiences^35–37^. Persons presenting with anorexia also showed altered experiences of embodiment^38,39^. Because explanatory models of the RHI considered it a pure perceptual illusion^4^, data from psychopathological populations has always been interpreted to reflect a different sensory-motor integration; a proof that senses work differently in those conditions^1,37^. However, our results may suggest a different process. Because the RHI is correlated to specific personality traits in healthy people, the relation with psychopathological conditions might be bidirectional.

On a similar line, Lush et al.^11^ recently published a study on a large sample (N = 353) where they found an association between the RHI and hypnotisability. The authors concluded that the key measures in the field of embodiment are, at least partially, driven by phenomenological control. While important methodological differences separate our two studies, they both converge to a similar conclusion. The RHI would not be a mere perceptual illusion, but a more complex phenomenon, where individual traits play a more central role than previously considered, and contextual factors should be considered cautiously when assessing the embodiment.

## Conclusions

With the present study, we propose a new scale (i.e., a shortened and reorganised version of the items of Longo et al.^15^) to measure the RHI. The new scale has a simple and clear structure with its three facets that showed good psychometric properties. The adoption of this scale will ensure the use of a questionnaire in the RHI based on solid empirical evidence. The ES already suggested new theoretical implications like the relation between embodiment and disembodiment. It also opened the field for new investigations, such as studying the conditions that may alter the physical sensations felt during the procedure. Finally, the relations observed with the self-esteem and the fantasy scale of empathy suggests reconsidering the RHI procedure in a new, broader, light. The RHI would not be a mere perceptual illusion, but the induced effect of embodiment would integrate with the complexity of the individuals and their differences.

## Methods

### Participants

Two-hundred-ninety-eight participants (221 females and 77 males, mean age 23.92, SD 4.05, range 18–55, years of education = 15.77, SD 1.60, range 13–18) took part in the study. Participants were right-handers (self-report), and naïve as to the purpose of the experiment.

They gave informed consent before being enrolled in the study, which was approved by the Ethical Committee of the University of Milano-Bicocca and conducted in accordance with the guidelines of the ethical standards of the Declaration of Helsinki^40^.

### Procedure

#### RHI procedure

**Figure 8:**
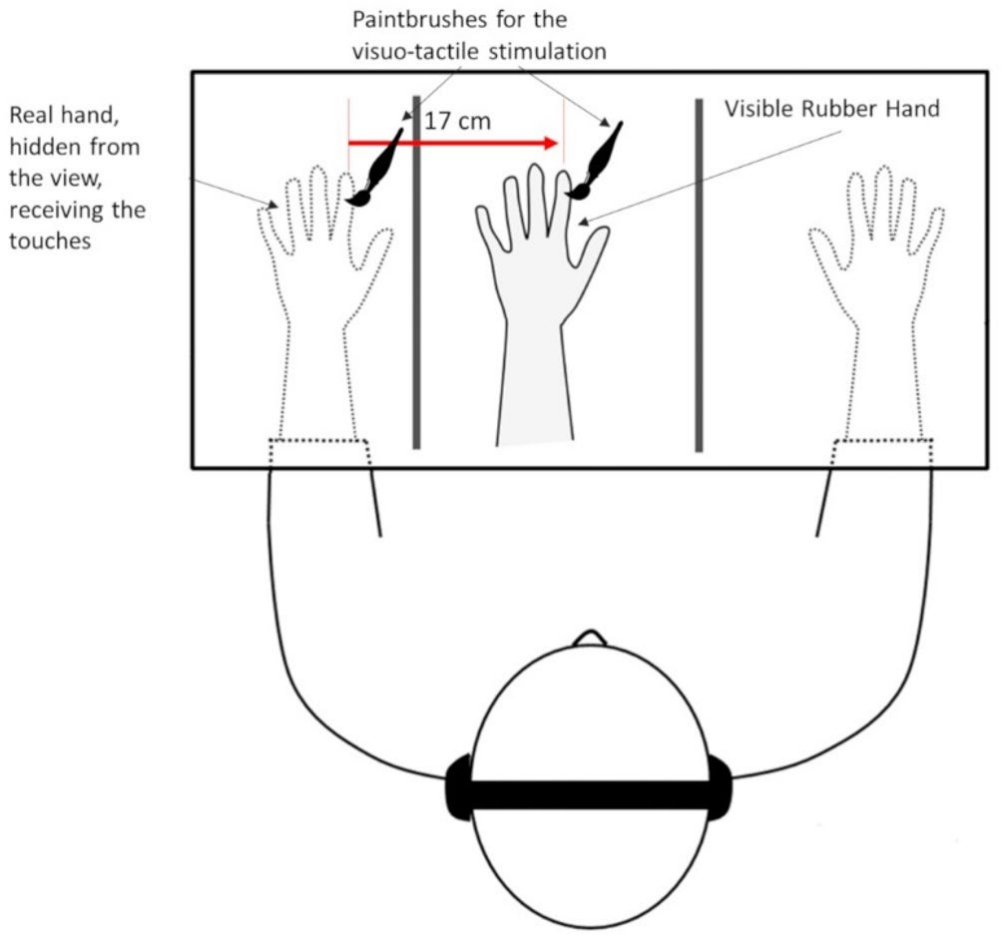
The Rubber Hand Illusion setup is depicted. Only the central compartment was visible during the illusory stimulation. The real hands were not visible during the illusion induction.

Participants sat in front of a table where a three-compartment steel box was placed. The participant's left hand was positioned in the left-side compartment with the index finger aligned with a fixed point (palm down). A realistic, left-sided, rubber hand was placed in an anatomically plausible position in the central compartment. The rubber hand was at a fixed distance of 17 cm towards the body midline respect the real left hand. The right hand rested palm-down in the third compartment (the one on the right) and was never visible during the entire experimental procedure. Wood panels separated the compartments. A black towel was laid upon the participant's shoulders and covered the space between his/her upper body and the frame (Fig. 8). A semi-transparent mirror topped the box aimed at occluding both the real and the prosthetic hands from sight during the assessment phase. A system of LED lights selectively allowed seeing below the semi-transparent mirror, so that the hands were visible, or not. In the assessment phase, the upper part of the structure was enlightened; by doing so, the top surface mirrored everything, preventing to see below the hands below the mirror surface.

During the stimulation phase, the upper lights were switched off, while the central compartment was lit by a set of LED lights arranged underneath the mirror, making visible only the rubber hand. Participants were instructed to look at the rubber limb in this phase. A trained experimenter stroked the participants real left hand and the rubber hand with two identical paintbrushes. Each participant underwent two conditions of stimulations. In the synchronous stroking condition, strokes were spatio-temporally congruent (i.e., the same finger was touched at the same time on both the real and the rubber hand). In the asynchronous stroking condition, touches on the real and fake hand were incongruent (i.e., different for both the location and/or the timing). In both conditions, the pattern of stimulation was unpredictable along the whole dorsum of the hands and fingers. The stimulation was manually delivered by multiple trained experimenters who were blind to the study's aims (i.e., master students doing an internship). The stimulation phases lasted 90 s. After each stimulation phase, participants completed the embodiment questionnaire. Half of the participants did the synchronous stimulation first, and half did the asynchronous first.

Personality questionnaires were completed at the end of the experimental session in a fixed order (IRI, FFMQ, RSE).

### Questionnaires

#### Embodiment Questionnaire (EQ)

The embodiment questionnaire^15^ included 27 items focused on the possible sensations felt during the rubber hand illusion experimental procedure. Each participant was administered two times the EQ, one after each condition of stimulation. The full list of items is reported in Table 1.

#### Interpersonal Reactivity Index (IRI) (Davis 1980)

The Interpersonal Reactivity Index (IRI) is a self-report questionnaire aimed at the explicit measurement of empathy. This tool was created to provide a multidimensional measure of empathy^17^. In the IRI participants had to complete 28 items via a Likert scale ranging from 0 ("Does not describe me well") to 4 ("Describes me very well"). The questionnaire is composed of four subscales of seven items each:

- Fantasy scale (FA), which reflects the tendency to project oneself into imaginary situations through the imagination.
- Perspective-taking (PT), which reflects the propensity to change perspective, to identify with another person adopting his/her viewpoint. PT refers to real-life events and everyday life and not fictitious situations.
- Empathic concern (EC), which measures the degree to which a person feels the negative experiences of another person feeling. It refers to warmth, compassion, and concern about others' emotions.
- Personal-distress (PD), which measures personal feelings of fear, worry, and discomfort in the face of negative experiences and misadventures that others are facing.

#### Five Facet Mindfulness Questionnaire (FFMQ)

The FFMQ is a questionnaire aimed at exploring the mindfulness construct and consists of 39 items that can be answered on a graduated scale (from 1 = "it never or very rarely happens to me" to 5 = "it happens to me often or always").

The FFMQ encompasses five subscales^18,41^.

- Non-react, which captures the attitude to avoid reacting to inner experiences.
- Observe, measuring the ability to observe, notice, and look after sensations, perceptions, thoughts and feelings emerging from one's inner experience.
- Act with awareness, a measure of the ability to act with focus and absence of distraction.
- Describe, measuring the ability to describe and label sensations, perceptions, thoughts, and feelings emerging from one's inner experiences in words.
- Non-judge, measuring the non-judgmental attitude towards one's inner experience.

#### Rosenberg Self-Esteem Scale (RSES)

RSES was developed to measure explicit self-esteem^19^. The scale was intended to be mono-dimensional and consists of 10 items containing 5 positive affirmations and 5 negative affirmations. Participants provide a graded response (from 1 = "strongly disagree" to 4 = "strongly agree ").

The scale measures one's global self-worth and includes aspects related to self-derogation (i.e., a defence of positive self-images against any threat) and self-enhancement (i.e., rise in the eyes of others).

### Research goals and analysis plan

#### Best solution of component structure of the RHI

We performed a PCA of the 27 items collected after synchronous stimulation. The number of factors was determined considering multiple indices. Specifically, we considered several criteria: the eigenvalues > 1, the parallel analysis, the Velicer's Minimum Average Partial—MAP (i.e., a method that suggests the model that best fits the data), the scree-test, and the interpretability of component contents. Solutions were Oblimin-rotated to allow for correlated components.

#### Hierarchical emergence of embodiment structure in the RHI

We explored subsequent solutions from the 3-component model, up to a 7-component model, which corresponds to the most complex sustainable solution (justified by eigenvalues > 1), using a Bass–Ackwards procedure^21^.

Bass–Ackwards calculates component scores for each of the desired solutions, then calculates the correlation between component scores, increasing by one the complexity of the solution. For example, the component scores of the 3-component solution are correlated with the component scores of the 4-component solution, whereas the component scores of the 4-component solution are correlated with the component scores of the 5-component solution. Following this procedure, we can explore and understand how different components emerge from simpler to more complex solutions.

#### The component structure of embodiment in the control condition

The comparison of results from the same items in two different conditions would require that the items' underlying structure is similar, provided that the data afford this possibility. One could argue that a questionnaire is thought to measure a construct (e.g., the embodiment) rather than to reflect the participant's behaviour in a specific experimental condition (e.g., the synchronous stimulation). Therefore, establishing similar structures for the synchronous and asynchronous conditions is essential to allow a meaningful comparison between the respective scores. Thus, we followed an exploratory PCA of the 27 items after asynchronous stimulation, with the Bass-Ackwards procedure on the same items, exploring solutions from the 3-component to a 7-component, as we did for the synchronous stimulation.

#### The Embodiment Scale (ES)

Based on the results of the PCAs, we propose an Embodiment Scale, a restricted version of Longo's questionnaire by selecting only the items that work efficiently in measuring the experience elicited by the RHI. We defined an item as working well enough as follows:

- it must have a strong primary loading (i.e., the correlation of the item with the component) > 0.4
- it must not have cross-loadings (any other loading must be < 0.25).

These are reasonable values that allow identifying items with sufficient primary loading while eliminating items with cross-loadings, therefore selecting the best markers of the components^42^.

After having selected the items, we run another PCA on the selected items to ascertain that the proposed scales and structure are psychometrically adequate.

We also evaluated the component congruence between the three-component solution for synchronous and asynchronous stimulation to confirm that the structure is similar between the two solutions. To do so, we calculated the Tucker's phi between synchronous and asynchronous solutions. Tucker's phi congruence coefficient is an index of component solution similarity calculated by correlating the two loading matrices^43^.

Once established the component similarity of the structures, we calculated the scale scores averaging the values of the items loading on each component.

The goodness of the factorial structure was furtherly ascertained by performing confirmatory factor analyses (CFA) on the selected items with the Lavaan package (version 0.6-6). Confirmatory factor analysis gives many indices that need to be evaluated in parallel to drive proper conclusions^24–26^.

The p value should be interpreted bearing in mind that non-significant values indicate a good fit. Notably, the p value is not the only fit indices to consider and it is actually affected by sample size. Thus, other indices are used to assess the goodness of fit of the model. For the relative fit indices TLI and CFI, values greater than 0.90 are considered as good and greater than 0.95 as very good. For the absolute measure of fit RMSEA and SRMR values lower than 0.08 are indicators of good fit^24–26^.

Finally, we calculated Cronbach's alpha and McDonald's omega to measure the internal consistency of the scales.

We provided descriptive statistics of scale scores at the group level. We then compared the average level of the two conditions with three paired samples t tests to verify which subscale is sensitive to the different stimulation congruency of synchronous and asynchronous RHI.

#### Individual differences in embodiment

We first calculated the RHI effect, which can be defined as the difference between the synchronous and asynchronous, namely the experimental and the control conditions. We thus calculated the delta scores for each scale. Then, we correlated delta scores with the scores of the IRI, FFMQ and RSE questionnaires.

Subsequently, we deepened the investigation with a network analysis. We used a Gaussian Graphical Model (GGM), in which edges correspond to partial correlation coefficients. GGM returns the association left between any two variables after conditioning on all other variables^20^. The GGM employs a regularisation penalty (i.e., the "least absolute shrinkage and selection operator"—LASSO^22^) that set small connections to zero^44^. The rationale behind the LASSO is to give back a conservative and parsimonious network with robust estimates^23^. The LASSO utilises a tuning parameter to control the degree to which regularisation is applied, selecting the parameter that minimises the extended Bayesian Information Criterion (eBIC)^45^. The tuning parameter for the model selection was set to a standard 0.5 in the current study. The issue of correcting for multiple comparsions in network analysis is still unresolved, but it comes into play when testing significant differences among paths, which we did not do^45^.

## Supplementary Information

Supplementary Information

## References

[1] de Vignemont F (2011). Embodiment, ownership and disownership. Conscious. Cogn..

[2] Romano D, Maravita A (2019). The dynamic nature of the sense of ownership after brain injury. Clues from asomatognosia and somatoparaphrenia. Neuropsychologia.

[3] Botvinick M, Cohen J (1998). Rubber hands ’feel’touch that eyes see. Nature.

[4] Tsakiris M (2010). My body in the brain: A neurocognitive model of body-ownership. Neuropsychologia.

[5] Lloyd DM (2007). Spatial limits on referred touch to an alien limb may reflect boundaries of visuo-tactile peripersonal space surrounding the hand. Brain Cogn..

[6] Costantini M, Haggard P (2007). The rubber hand illusion: Sensitivity and reference frame for body ownership. Conscious. Cogn..

[7] Tsakiris M, Carpenter L, James D, Fotopoulou A (2009). Hands only illusion: Multisensory integration elicits sense of ownership for body parts but not for non-corporeal objects. Exp. Brain Res..

[8] Marotta A, Tinazzi M, Cavedini C, Zampini M, Fiorio M (2016). Individual differences in the rubber hand illusion are related to sensory suggestibility. PLoS One.

[9] Walsh E, Moore JW, Oakley DA, Halligan PW (2015). Are you suggesting that ’ s my hand? The relation between hypnotic suggestibility and the rubber hand illusion. Perception.

[10] Haans A, Kaiser FG, Bouwhuis DG, Ijsselsteijn WA (2012). Individual differences in the rubber-hand illusion: Predicting self-reports of people’s personal experiences. Acta Psychol. (Amst.).

[11] Lush P (2020). Trait phenomenological control predicts experience of mirror synaesthesia and the rubber hand illusion. Nat. Commun..

[12] Makin TR, Holmes NP, Ehrsson HH (2008). On the other hand: Dummy hands and peripersonal space. Behav. Brain Res..

[13] DAlonzo M, Clemente F, Cipriani C (2014). Vibrotactile stimulation promotes embodiment of an alien hand in amputees with phantom sensations. IEEE Trans. Neural Syst. Rehabil. Eng..

[14] Bolognini N, Ronchi R, Casati C, Fortis P, Vallar G (2014). Multisensory remission of somatoparaphrenic delusion: My hand is back!. Neurol. Clin. Pract..

[15] Longo MR, Schüür F, Kammers MPM, Tsakiris M, Haggard P (2008). What is embodiment? A psychometric approach. Cognition.

[16] Walsh E (2015). Are you suggesting that’s my hand? The relation between hypnotic suggestibility and the rubber hand illusion. Perception.

[17] Davis MH (1980). A multidimensional approach to individual differences in empathy. Cat. Sel. Doc. Psychol..

[18] Giovannini C (2014). The Italian Five Facet Mindfulness Questionnaire: A contribution to its validity and reliability. J. Psychopathol. Behav. Assess..

[19] Rosenberg M (1979). Conceiving the Self.

[20] Costantini G (2015). State of the aRt personality research: A tutorial on network analysis of personality data in R. J. Res. Pers..

[21] Goldberg LR (2006). Doing it all Bass-Ackwards : The development of hierarchical factor structures from the top down. J. Res. Personal..

[22] Zou H (2006). The adaptive lasso and its oracle properties. J. Am. Stat. Assoc..

[23] Lu W, Goldberg Y, Fine JP (2012). On the robustness of the adaptive lasso to model misspecification. Biometrika.

[24] Gana K, Broc G (2019). Structural Equation Modeling with Lavaan.

[25] Hu LT, Bentler PM (1998). Fit indices in covariance structure modeling: Sensitivity to underparameterized model misspecification. Psychol. Methods.

[26] Kline RB (2015). Principles and Practice of Structural Equation Modeling.

[27] Giummarra MJ, Gibson SJ, Georgiou-Karistianis N, Bradshaw JL (2008). Mechanisms underlying embodiment, disembodiment and loss of embodiment. Neurosci. Biobehav. Rev..

[28] Newport R, Preston C (2010). Pulling the finger off disrupts agency, embodiment and peripersonal space. Perception.

[29] Newport R, Gilpin HR (2011). Multisensory disintegration and the disappearing hand trick. Curr. Biol..

[30] Moseley GL (2008). Psychologically induced cooling of a specific body part caused by the illusory ownership of an artificial counterpart. PNAS.

[31] Barnsley N (2011). The rubber hand illusion increases histamine reactivity in the real arm. Curr. Biol..

[32] della Gatta F (2016). Decreased motor cortex excitability mirrors own hand disembodiment during the rubber hand illusion. Elife.

[33] Folegatti A, Farnè A, Salemme R, de Vignemont F (2012). The Rubber Hand Illusion: Two’s a company, but three’s a crowd. Conscious. Cogn..

[34] Rohde M, Di Luca M, Ernst MO (2011). The rubber hand illusion: Feeling of ownership and proprioceptive drift do not go hand in hand. PLoS One.

[35] Lev-Ari L, Hirschmann S, Dyskin O, Goldman O, Hirschmann I (2015). The Rubber Hand Illusion paradigm as a sensory learning process in patients with schizophrenia. Eur. Psychiatry.

[36] Rossetti I (2020). Defective embodiment of alien hand uncovers altered sensorimotor integration in schizophrenia. Schizophr. Bull..

[37] Asai T, Mao Z, Sugimori E, Tanno Y (2011). Rubber hand illusion, empathy, and schizotypal experiences in terms of self-other representations. Conscious. Cogn..

[38] Zopf R, Contini E, Fowler C, Mondraty N, Williams MA (2016). Body distortions in Anorexia Nervosa: Evidence for changed processing of multisensory bodily signals. Psychiatry Res..

[39] Keizer A, Van Elburg A, Helms R, Dijkerman HC (2016). A virtual reality full body illusion improves body image disturbance in anorexia nervosa. PLoS One.

[40] World Medical Organization (1996). Declaration of Helsinki. Br. Med. J..

[41] Baer RA, Smith GT, Hopkins J, Krietemeyer J, Toney L (2006). Using self-report assessment methods to explore facets of mindfulness. Assessment.

[42] Gorsuch RL, Nesselroade JR, Cattell RB (1988). Exploratory factor analysis. Handbook of Multivariate Experimental Psychology. Perspectives on Individual Differences.

[43] Lorenzo-Seva U, Ten Berge JM (2006). Tucker’s congruence coefficient as a meaningful index of factor similarity. Methodology.

[44] McNeish DM (2015). Using lasso for predictor selection and to assuage overfitting: A method long overlooked in behavioral sciences. Multivar. Behav. Res..

[45] Epskamp S, Borsboom D, Fried EI (2018). Estimating psychological networks and their accuracy: A tutorial paper. Behav. Res. Methods.

