# Supplementary material for: Psychometric properties of the embodiment scale for the rubber hand illusion and its relation with individual differences
Source: Sci Rep. 2021 Mar 3;11:5029. doi: 10.1038/s41598-021-84595-x (PMC7930179; doi:10.1038/s41598-021-84595-x)
Supplement: Supplementary file 1 — Supplementary Information [file 41598_2021_84595_MOESM1_ESM.docx]

# Title: Psychometric properties of the Embodiment Scale for the rubber hand illusion and its relation with individual differences.

# Authorship: Daniele Romano^1,2,3 *^, Angelo Maravita^1^, Marco Perugini^1,2^

**Affiliations**:

^1^ Psychology Department, University of Milano-Bicocca

^2^ Mind and Behavior Technological Center (MIBTEC), University of Milano-Bicocca

^3^ Department of History, Society and Human Studies, University of Salento

**Supplementary material:**

1. Embodiment Scale.
2. Decomposition of the Embodiment component
3. Bass Ackwards solutions. Full description.

**A) Embodiment Scale**.

**Embodiment Scale (ES)**

From -3 to +3, how much do you agree with the following statements.

**“During the block...**

1(E1) ...it seemed like I was looking directly at my own hand, rather than at a rubber hand -3 -2 -1 0 +1 +2 +3 _______

2(E2) ...it seemed like the rubber hand began to resemble my real hand -3 -2 -1 0 +1 +2 +3 _______

3(E3) ...it seemed like the rubber hand belonged to me -3 -2 -1 0 +1 +2 +3 _______

4(E4) ...it seemed like the rubber hand was my hand -3 -2 -1 0 +1 +2 +3 _______

5(E5) ...it seemed like the rubber hand was part of my body -3 -2 -1 0 +1 +2 +3 _______

6(E6) ...it seemed like my hand was in the location where the rubber hand was -3 -2 -1 0 +1 +2 +3 _______

7(E7) ...it seemed like the rubber hand was in the location where my hand was -3 -2 -1 0 +1 +2 +3 _______

8(E8) ...it seemed like the touch I felt was caused by the paintbrush touching the rubber hand -3 -2 -1 0 +1 +2 +3 _______

9(E9) ...it seemed like I could have moved the rubber hand if I had wanted -3 -2 -1 0 +1 +2 +3 _______

10(E10) ...it seemed like I was in control of the rubber hand -3 -2 -1 0 +1 +2 +3 _______

11(D1) ...it seemed like I was unable to move my hand -3 -2 -1 0 +1 +2 +3 _______

12(D2) ...it seemed like I couldn’t really tell where my hand was -3 -2 -1 0 +1 +2 +3 _______

13(D3) ...it seemed like my hand had disappeared -3 -2 -1 0 +1 +2 +3 _______

14(D4) ...it seemed like my hand was out of my control -3 -2 -1 0 +1 +2 +3 _______

15(D5) ...it seemed like my hand was moving towards the rubber hand -3 -2 -1 0 +1 +2 +3 _______

16(D6) ...it seemed like the rubber hand was moving towards my hand -3 -2 -1 0 +1 +2 +3 _______

17(P1_r) ...the touch of the paintbrush on my ﬁnger was pleasant -3 -2 -1 0 +1 +2 +3 _______

18(P2) ...I had the sensation of pins and needles in my hand -3 -2 -1 0 +1 +2 +3 _______

**E – embodiment** = _____________ (E1+E2+E3+E4+E5+E6+E7+E8+E9+E10)/10

**D – disembodiment** = __________ (D1+D2+D3+D4+D5+D6)/6

**P – physical sensations** = ________ (P1_r*-1^#^ + P2)/2

^#^ the item P1_r can be simply subtracted instead of added in calculating the mean. The resulting formula is P = (P2 - P1_r)/2

**B) Decomposition of the Embodiment component**

Longo et al.’s (2008) further differentiated their general component of Embodiment by performing PCAs only on the items grouped by this general component (first ten items). As an ancillary aim, we performed a PCA analysis on the first ten items, in the effort of replicating Longo’s results at this fine-grained level of specificity ^14^.

We run an exploratory PCA on the ten items, and then we followed it with a BA procedure exploring solutions from the 1-component to the theoretical three-components. This procedure was repeated for both synchronous and asynchronous items.

*Results*

## The PCA on the first ten items of synchronous stimulation shows that three components had eigenvalues > 1, with the scree-plot and MAP suggesting a single component, and the parallel analysis two-components.

The single -component solution works well and explains 58% of the variance, with all items loading above .60, except Item 7 which anyway showed a sizeable loading of .50.

The two-component solution explains 68% of the variance. After an Oblimin rotation (components are correlated 0.37), the first component can be interpreted as an embodiment component gathering items about ownership (items 1:5 loadings from .62 to .80) and agency (9 and 10, loading .92 each) whereas the second is about the location (items 6, 7 loadings .6 and .97).

In the three-component solution, most items loaded as expected by Longo et al.’s (2008). The first five items on a component Ownership (loadings from .69 to .89), item 6 and 7 on Location (.60 and .97), item 9 and 10 on Agency (.92 and .91). Item 8 did not load as expected on the Location component but rather loads on Ownership (.63). The components strongly correlate with each other (from .31 to .65). The solution explains 76% of the variance.

## The PCA on the same items in the Asynchronous stimulation condition shows that one component only had eigenvalues > 1 and all other criteria converged on indicating one component as the better solution.

The single component solution explains 65% of the variance with all items showing high loadings (from .69 to .91).

The two-component solution represents an embodiment component that clusters together ownership and location (items 1:8 loadings from .54 to .92) and an agency component (items 9 and 10, loading .88 and .91). The two components are highly correlated (.59) and explain together 74% of the variance.

In the three-component solution, the items loaded following the theoretical expectations, in line with the results in the Synchronous condition. Component 1 is about Ownership (item 1 to 5 from .87 to .97), component 2 is about Agency (items 9 and 10, loading .92 and .96), and component 3 is about Location (items 6 and 7, loading .82 and .99). Item 8 is spread over the three components but with insufficient loadings (the highest is .30). The components are highly correlated (from .46 to .62), and the solution explains 82% of the variance.

*Conclusions*

For both conditions, our data clearly suggest a single component solution. However, when a three-component solution is extracted, the results go in the expected direction and the items clusters in the same way as found by Longo’s et al. in the Ownership, Location and Agency components, with the exception of item 8.

What does this mean? Should we split the items in a more complex structure or not? Our data suggests we should not. However, when we created the ES, we paid attention to capture the entire breadth of the Embodiment and Disembodiment components. This means that items about ownership location and agency were kept in the Embodiment subscale, as well as Movement and Loss-of-own-hand items were kept in the Disembodiment subscale.

**C) Bass Ackwards solutions. Full description.**

*Synchronous Stimulation*

We explored subsequent solutions from the 3-component model, which corresponds to our best solution, up to a 7-component model, which corresponds to the most complex sustainable solution (justified by eigenvalues >1, see below), using a Bass-Ackwards procedure ^21^.

The three-component solution (fully described in the main text) is the best solution and identifies one component capturing the Embodiment of the fake hand, one related to the Disembodiment of one's hand, and one capturing the Physical sensations experienced.

The four-component solution presents once again three clear components related to the embodiment (1:10), disembodiment (12:18) and physical sensation (22, 23, 24, 27), while the fourth component is defined insufficiently, with only one item clearly loading on it (19).

The five-component solution presents one compact component gathering items about the embodiment of the rubber hand (1:10). The disembodiment component splits in two, items from 12 to 16 load on component two and are all related to the experience of losing one's own hand. Items 17 and 18 loaded maximally on component four. They referred to movement sensations. Component three resumes items about physical sensations (items 22, 23, 24, 27). Finally, component five is again insufficiently defined with only one item unequivocally loading on it (item 20), which is, by the way, different from the four-component solution.

The six-component solution identifies the component about embodiment sensations (1:10), which however seems less compact, as a few items spread over component six (items 6 and 7). The sixth component is unclear as it collects items about the location of the fake hand (items 7 and in part item 6), but also item 19 which referred to the experience of having three hands. The two components of disembodiment remained split in loss-of-own-hand (12:16) and movement sensations (17, 18). The physical sensation component remains clearly identifiable in component three, with the maximal loadings of items 22 and 23. Differently from the previous solution, here emerges more clearly a component related to the pleasantness of the experimental experience in component five, similar to the one that Longo called "affect" (items 20 and 21).

The seven-component solution keeps the first component about embodiment with items 6 and 7 still spreading on a different component (component 7) which remains unclear as it still gathers items 7, 19 and item 6 cross-loading on component 1. Like previous solutions, we identified the component affect (items 20, 21), physical sensation (items 22, 23,24,27), and movement (items 17,18). The remaining two components split items as follows: items 12, 13 and 16 loaded on component 4 and referring to the sensation of losing control of one's hand; items 14 and 15 loaded on component five and referring to the sensation that the physical hand disappeared.

Correlations between components scores of different level solutions are depicted in Figure 2 of the main text and reported here for easiness of reading.


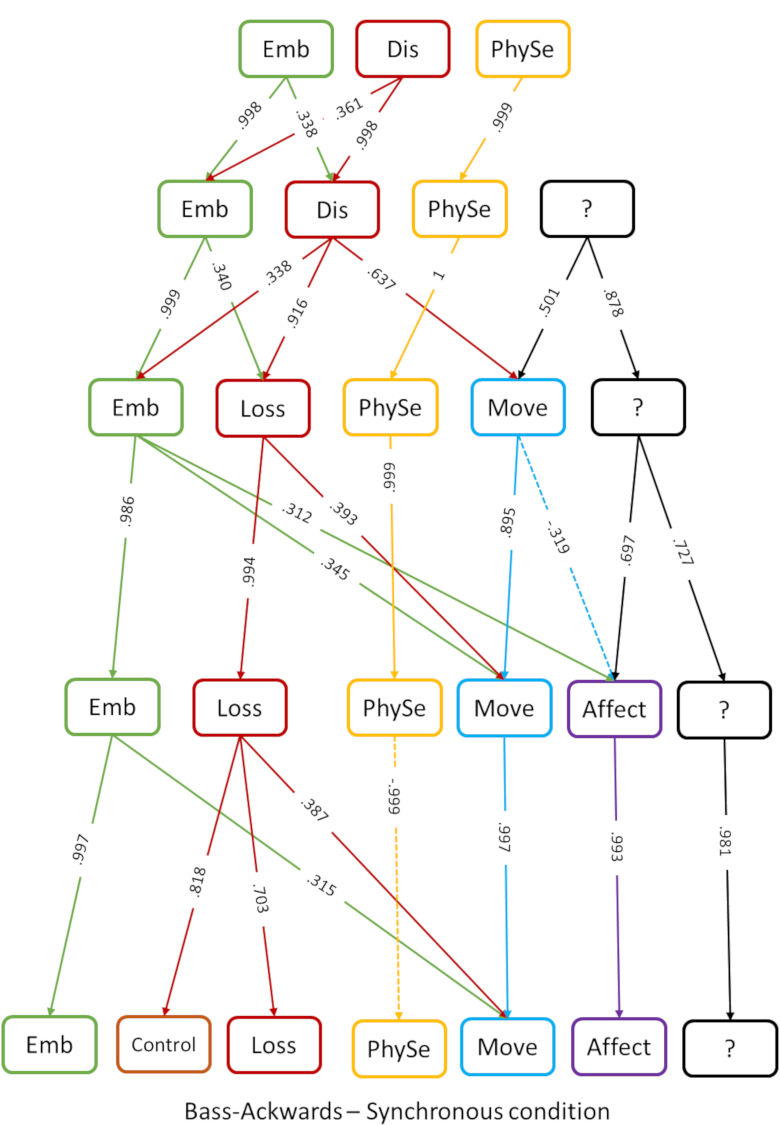


**Figure S1.** Results of the Bass-Ackwards procedure on the items after synchronous stimulation. The correlations between the components of different solutions are represented. Negative correlations are reported with dash lines, while positive correlations are in solid lines. Components’ names are abbreviated: Emb=embodiment, Dis=disembodiment, PhySe= Physical Sensations, Loss= loss-of-own-hand, Move= movement, Affect= affect towards the general experience, Control= control on own hand actions, ?= undefined component. Colours are used to help the readability of the graph.

In general, the first component remains substantially the same across all solutions. We never clearly recovered the three subcomponents ownership, agency and location. The disembodiment component splits in the two-component observed by Longo' study, increasing the complexity of the structure. They emerge as independent components in the five-component solution.

Notably, we identified a component about physical sensations experienced on one's hand already in the three-component solution. This component remains unaffected by the increasing complexity with correlations >.98 from one solution to another. This component is similar, although not identical, to the one described by Longo as "deafference".

*Asynchronous Stimulation*

The three-component solution shows a component 1 loaded by items about the embodiment of the rubber hand (1:10) plus item 24. Component 2 gathers items about disembodiment of one's hand (12:18). Component 3 focused on items 22 and 23, and we may refer to it again in terms of physical sensations. Overall, the three-component solution seems remarkably similar to the one obtained following synchronous stimulation.

The four-component solution presents one component related to the embodiment (1:10), one component collecting items about the loss of one's hand (12:16), one component related to the sensation of movement of the hands (17, 18 and in part also 19), the fourth component about physical sensation (22, 23). Notably, item 24 loaded on component one in the asynchronous stimulation.

The five-component solution presents one component gathering items about the embodiment of the fake hand (1:10) again. The loss of hand component (12, 14, 15, 16) remains clearly identifiable in component 2, as well as the component movement (17, 18, 19) in component 3. Component four have maximal loadings by items 22 and 23, keeping the main core of physical sensations. Component five emerges as new in putting together items 20 and 21, and we can refer to it as affect to the general experience.

The six-component solution maintains the component about embodiment (1:10). Component two remains anchored to the items about hand loss (12, 14, 15, 16). Component three is focused around items 17, 18, and in part 19, those about movement. Component four is about physical sensations gathering the items 22 and 23. Component five cluster together items 20 and 21 referring to the pleasantness of the general experience similar to the affect component individuated by Longo previously. The sixth component is unclear, only item 25 (about vividness) undoubtedly loaded on it, while a consistent loading, although in the presence of a strong cross-loading, was detectable also for item 13 (about control). A clear interpretation is not self-evident for the component.

The seven-component solution looks more confusing in terms of interpretability of many components. Component one remains anchored around embodiment items (1:10). However, a few of them spread their loadings on other components (items 6 and 7 on component six; item 8 on component seven). Component 2 is still concerning the items of hand' loss (12:16). Component 3 captures the movement sensations (17, 18). Component 4 refers to physical sensations again with maximal loadings by items 22 and 23. Component 5 is about affect (items 20 and 21). Component six and seven look difficult to interpret. Component six has only two items that primarily load on it; item 25 and item 7, which however has a strong cross-loading on component 1. Similarly, component seven has items 8 (referred touch) and 24 (numbness) that primarily load on it, with item 8 that cross-loaded on component 1.

Correlations between components of subsequent solutions obtained with the Bass-Ackwards are represented in Figure 4 of the main text and reported also here for easiness of reading.


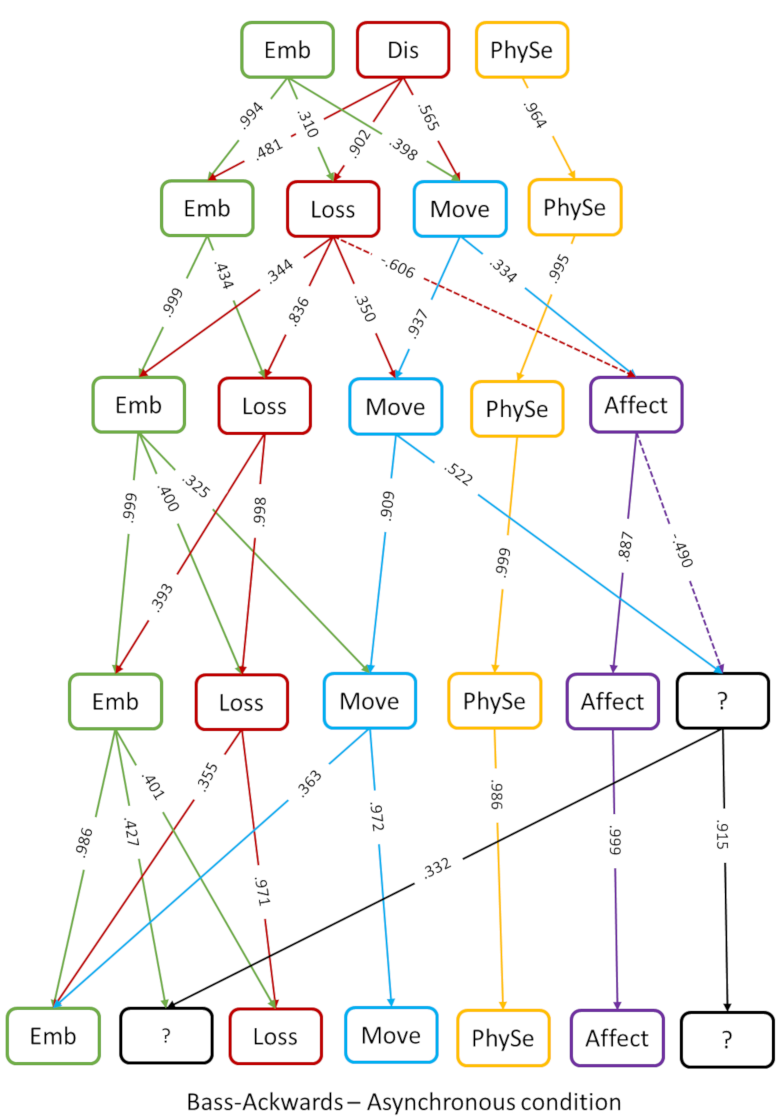


**Figure S2.** Results of the Bass-Ackwards procedure on the items after asynchronous stimulation. The correlations between the components of different solutions are represented. Negative correlations are reported with dash lines, while positive correlations are in solid lines. Components’ names are abbreviated: Emb=embodiment, Dis=disembodiment, PhySe= Physical Sensations, Loss= loss-of-own-hand, Move= movement, Affect= affect towards the general experience, Control= control on own hand actions, ?= undefined component. Colours are used to help the readability of the graph. .

To summarise, also in the asynchronous condition, we were unable to distinguish any subcomponent of embodiment. The correlations between components showed that the component embodiment and physical sensations remain constant in all solutions. The six and seven components solutions seem to be a not good explanation of our data resulting in almost meaningless components. In the five-component solution, the component affect emerges almost as a new component and is only slightly correlated with the movement component of the four-component solution. The four-component solution, as compared to the three-component solution, does not introduce anything radically different, but it simply offers a finer distinction of one of the three components.
